# Heart Rate Variability Assessment of Land Navigation and Load Carriage Activities in Specialist Police Selection

**DOI:** 10.3390/healthcare11192677

**Published:** 2023-10-03

**Authors:** Colin D. Tomes, Elisa F. D. Canetti, Ben Schram, Robin Orr

**Affiliations:** 1 Faculty of Health Science and Medicine, Bond University, Robina, QLD 4226, Australia; ecanetti@bond.edu.au (E.F.D.C.); bschram@bond.edu.au (B.S.); 2 Tactical Research Unit, Bond University, Robina, QLD 4226, Australia

**Keywords:** SWAT, HRV, biomarkers, data visualization, stress, attrition

## Abstract

Police tactical group (PTG) personnel are exposed to physical, mental, and emotional stressors. Consequently, PTG selection courses (SCs) impart similar challenges, often resulting in candidate attrition. Holistic assessment may provide additional support to stakeholders given these risks. Heart Rate Variability (HRV) is an objective holistic stress measure that may be applicable in PTG SCs but has not been thoroughly researched. Therefore, this study aimed to report HRV data in an end-user accessible format and determine the relationship between HRV and attrition. A total of 18 qualified Australian State law enforcement officers completed a 1-day physical readiness assessment. Of those, six males progressed to an additional two-day course, on which this study is focused. This two-day selection consisted of additional physical challenges and occupational assessments. HRV was obtained from 2-lead ECGs and defined as the percentage of R-R intervals that varied by ≥50 ms (pRR50). Data were summarized in a heat map of consecutive short-term analyses. Three candidates withdrew. A logistic regression based on heat map data found high HRV was significant for predicting attrition, χ^2^ (6) = 8.318, *p* = 0.004. HRV may provide insight for PTG stakeholders monitoring attrition. While the sample size was limited and replication is needed, this study tentatively establishes value for HRV monitoring in PTG SCs.

## 1. Introduction

Police work is known to expose officers to high levels of physical, mental, and emotional stressors [1,2]. Imposed demands include response to emergencies, internal organizational demands, irregular work hours or extended shifts, and potentially service as a specialist [3,4]. In Australia, Police Tactical Groups (PTGs) are one of the most demanding specialist capacities in which an officer can elect to serve [5,6]. The specialist police who serve in PTG units are subject to additional demands beyond those encountered by general duty police officers [7,8]. As such, the PTG officer may undertake a wide scope of duties that include search and rescue, counterterrorism, explosive ordinance disposal, high-risk warrant service, and policing engagements involving hostile entities with firearms [5,9].

To ensure candidate personnel are prepared to meet these occupational demands, the selection course to join a PTG unit is physically demanding and technically challenging. Highly refined marksmanship skills, excellent physical fitness, and an exceptional ability to perform under extreme pressure are all cardinal traits for success [10,11]. The rigor of specialist selection aims to ensure future team members are not only physically, but mentally and emotionally prepared for PTG service [3]. One notable and critical requirement of PTG service is the ability to carry heavy loads over extended timespans while still performing other critical duties. Whereas general duty police officers may wear and carry loads of around 10 kg [12], specialist police officers can carry loads of around 20 kg, increasing up to 40 kg or more when additional stores (such as breaching equipment and ballistics shields) are required [13]. Consequently, PTG selection courses include often arduous load carriage events [14,15]. Further, these tasks may be required in challenging environments, such as hot and humid conditions, which can exacerbate completion difficulty, physiological demand, and subjective perception of exertion [16,17,18,19].

In addition to physical competency, mental fortitude to cope with psychological stressors of the professions is a desirable characteristic expected of candidates [11]. With this in mind, psychometric testing, personal interviews, and expert observation of performance are also incorporated to selection courses, creating a holistic picture of a candidate’s overall suitability for the profession [20]. Often, events in the selection course will incorporate both physical and mental stressors, such as a prolonged load carriage event in a mentally challenging course under adverse conditions (e.g., a navigation exercise across various terrains). Monitoring the physiological impacts of these two types of stressors can provide decision makers additional information on the performance of personnel undergoing selection [21]. The refinement of selection courses and human performance monitoring may be especially critical as application pools continue to shrink [22], and police organisations face increasing scrutiny [23]. It may be possible that both through selection and across the career, health and human performance supports may be a valuable strategy for improving the health and safety of officers and communities [24].

Wearable technology has enhanced the capacity for health and performance support personnel to obtain physiological data from individuals in austere locations. Decreased HRV may not necessarily indicate worsened health or performance in all settings, and conversely, high HRV may not exclusively indicate high performance and optimal health [25]. However, in tactical settings where stress and sources of allostatic load are abundant and often extreme, low HRV may be more reasonably expected as an indicator of concern [25,26,27]. Examples of monitoring for depressed HRV include military basic training or advanced specialist training [21,26] as well as within firefighting units and professional rescuer teams [28,29,30]. One performance indicator obtainable from these wearables is Heart Rate Variability (HRV). HRV generally aims to computationally describe the dynamic, interdependent, and multisystem psychophysiological responses that modulate the time between consecutive heart beats, also known as beat-to-beat or inter-beat intervals (IBI) [31]. A dynamic relationship between intrinsic cardiac regulatory factors, as well as factors external to the heart responding to the environment, namely the sympathetic (SNS) and parasympathetic (PNS) nervous systems [31,32], drive fluctuations in IBI. Therefore, the use of HRV assessment is considered a viable measure for quantifying stress holistically in many career fields but may be particularly beneficial in tactical settings, as stress in tactical populations may be particularly intense and multifactorial [29,33,34]. When other strategies for evaluating autonomic regulation or stress response are inaccessible due to the nature of tactical work, HRV shows promise as a deployable measurement tool for identifying stress responses [2,35]. For these reasons, HRV monitoring has been utilized with increasing frequency in military [26], fire [36], and rescue populations [37]. However, its application in the specific context of PTG selection remains subject to limited inquiry [38]. Therefore, the aim of this study was to (1) pragmatically implement wearable HRV monitoring during prolonged load carriage events conducted during PTG selection, (2) provide data in an easily accessible format, and (3) determine if the data could be used to assess candidate attrition. The research question was as follows: can HRV data obtained during specialist police selection load carriage activities be processed into an accessible format for end-users and can those data predict attrition? The authors hypothesised that HRV would be practical, presentable in an efficient manner to key stakeholders, and that HRV would be sufficiently sensitive to distinguish candidates that withdrew from those that did not.

## 2. Materials and Methods

This study was a prospective cohort study of six male PTG candidates undergoing specialist selection at an Australian State PTG facility in Autumn of 2022. Female candidates were eligible both for selection and inclusion in this study, but none had applied for the selection cycle on which this report is based. Table 1 contains mean descriptive data for those anthropometrics and fitness data that could be released; further detail regarding these variables could not be published as per a privacy agreement with the sponsoring organisation. For this study, height was self-reported; self-reported anthropometrics have been utilized as reliable metrics in research in the previous literature in law enforcement populations [39].

Candidates were supplied with an Equivital™ EQ02+ LifeMonitor (Equivital, Cambridge, UK) wearable monitoring harness sampling at 256 Hz to capture ECG activity from the start of the selection course until the candidate was withdrawn or the selection course ended. Each harness was individually fitted to the level of each candidate’s xiphoid process to ensure electrode contact points were secured. Previous research indicates the Equivital system is comparable in terms of validity and reliability to the gold-standard Holter ECG monitor [40]. ECG data were captured with Equivital software (Equivital Manager 2.9.14.260, Cambridge, UK) and processed with LabChart version 8 (ADInstruments, Sydney, Australia). Visual ECG examination was performed in combination with automated detection of signal noise provided by the LabChart Software v8. ECG complexity was set between 1.0 and 1.5. Acceptable R-R intervals were established as those beats that fell between 272 and 1600 ms. This is equivalent to a heart rate between 220 bpm and 37.5 bpm. Any identified beats falling outside this range were checked manually and either included or excluded based on the visual features of the ECG. HRV was defined as the percentage of R-R intervals that varied by more than 50 ms (pRR50). Results were summarized in a heat map format consisting of 153 consecutive short-term analyses, each lasting 5 min. This reporting strategy allowed for the greatest resolution of HRV changes between tasks, without sacrificing validity, given potential for signal noise in this context. The total time span was from 0700 to 2000 h on the initial day of a two-day selection course. The end point of 2000 h was identified as the threshold time for suspending analysis, as three of the six candidates were medically withdrawn at approximately that time.

### 2.1. Selection Course and Load Carriage Events

The initial cohort was comprised of 18 male police officers who were inducted into a 1-day physical training selection assessment. Of those 18, six individuals were eligible to participate in an additional two-day selection course. The one-day course primarily screened for physical fitness and workload tolerance. Attrition was high, resulting in six of those 18 progressing to participate in an additional two-day selection course, which is the focus of this study. There were no exclusion criteria; all personnel eligible for the two-day assessment were eligible for study inclusion and recruited. In general, the two-day selection course consisted of additional physical training activities, but also essential specialist police task training and assessment activities. These included orienteering, firearms manipulation, threat de-escalation, load carriage, and casualty evacuation. Specifically for this study, HRV response to prolonged load carriage (~12 kg) was of chief interest as load carriage tasks are a fundamental requirement for these personnel [41] and may be associated with selection failure [14,42]. As such, the 13 h of continuous load carriage activities that took place during the overall selection and assessment process formed the focus of this work. These load carriage events included a land navigation exercise in which candidates were tasked with locating various landmarks from longitude and latitude only, utilizing simple tools such as a paper map, pencil, and compass. The land navigation load configuration consisted of self-selected combinations of standardised web belts, uniforms, modular pouches, and chest harnesses on which candidates mounted water, the land navigation equipment, and other tactical stores. The land navigation exercise took place in an undeveloped area with mostly level terrain. Environmental temperatures ranged from 24.2–34.4 °C and the relative humidity ranged from 60–75% (Australian Bureau of Meteorology). Landmarks were approximately 1–3 km apart from both the starting area and each other. Candidates were not told how many landmarks needed to be reached successfully to achieve a passing outcome. The other load carriage event consisted of a pack march on a level, paved road surface. Participants continued marching with no knowledge of the required time or distance.

### 2.2. HRV Outcome Measures

The pRR50 metric was chosen specifically because it has been identified as a clinically important feature when assessing for risk of cardiovascular disease (CVD). CVD is known to affect police officers to a greater extent than the general population [43,44,45]. This metric is also less volatile and more amendable to use in short-term recordings than frequency-domain measures [46]. While lower values of HRV are not exclusively indicative of worsened health or performance, and high values are not exclusively indicative of high performance and optimal health, in tactical settings where stress and sources of allostatic load are abundant and often extreme, low HRV may be more reasonably expected as an indicator of concern [25,26,27]. Specifically, a lower pRR50 value indicates less variation in IBI for the analysis period, and therefore decreased responsiveness to dynamic external and internal environmental fluctuations. Insufficient responsiveness to stimuli may in turn signal excessive physical or psychophysiological stress [32,47]. Consecutive 5 min short-term blocks, rather than one single long-term (13 h) analysis, was performed to allow for the greatest resolution of changes possible during the selection course without sacrificing validity. Heat map shading was informed by the work by Hopkins and Buchheit [48,49], using the mean and standard deviation (SD) of each participant’s pRR50 values. This controls for the highly individual nature of HRV and its volatility while still permitting meaningful analysis of the entire cohort [50]. Values in each short-term block above ½SD of the participant’s mean (for all blocks) were shaded green, within ±½SD of the participant’s mean were shaded yellow. Values below ½SD of the participant’s mean were shaded red.

### 2.3. Participants

Candidates were briefed by the research team to inform them that their decision to participate would in no way influence their selection result. This briefing was reiterated by the assessment selection staff. After agreeing to participate, all candidates disclosed that they were taking no medications for cardiovascular, renal, or respiratory conditions as a requirement of participation in this study. During selection activities, all candidates were provided uniform rations by the assessing organization. Water was provided ad libitum, but all other beverages, including caffeine, were restricted. All candidates provided their informed written consent, and the unit commander provided permission for publication of this work. The research protocol was approved by the Bond University Human Research Ethics Committee (BUHREC) (Protocol 2019-022 amnd 2) and all procedures were conducted in accordance with the Declaration of Helsinki of 1964 and its later amendments [51].

### 2.4. Statistical Analyses

Descriptive analytics were derived from the heat map. Visual inspection of histograms was conducted for each participant to ascertain distribution, skew, and kurtosis. These can be found in the Supplementary Figure S1. These included count data tabulations of total time and longest consecutive time span in each of the shaded color zones of the heat map. Given the small sample size, Mann–Whitney U tests were planned for direct comparison of raw HRV and heat map count data between those candidates that withdrew during the pack march and those that did not. Logistic regression analyses were also performed to determine if attrition could be predicted. The dichotomous variable for each model was selection success or failure (1 or 0). For continuous variables, models were attempted using raw HRV values and count data from the heat map analysis. Model iterations utilizing heat map count data were drawn from the beginning of the selection course (0700) to the end of the first load carriage activity, being land navigation (1430), and included the total quantity of shaded 5 min intervals with either ‘green’ shading representing >½SD from the mean value, ‘red’ shading <½SD from the mean value, or ‘yellow’ shading (the intermediary 1SD surrounding the mean). The time range was chosen to determine if HRV response during the first event would be predictive of attrition that occurred in the second event. All statistics were calculated using JASP 0.17.2.1 (JASP Project, University of Amsterdam, Amsterdam, The Netherlands). Sensitivity and specificity were calculated from successful (*p* < 0.05) models using the regression package found in JASP.

## 3. Results

**Figure 1:**
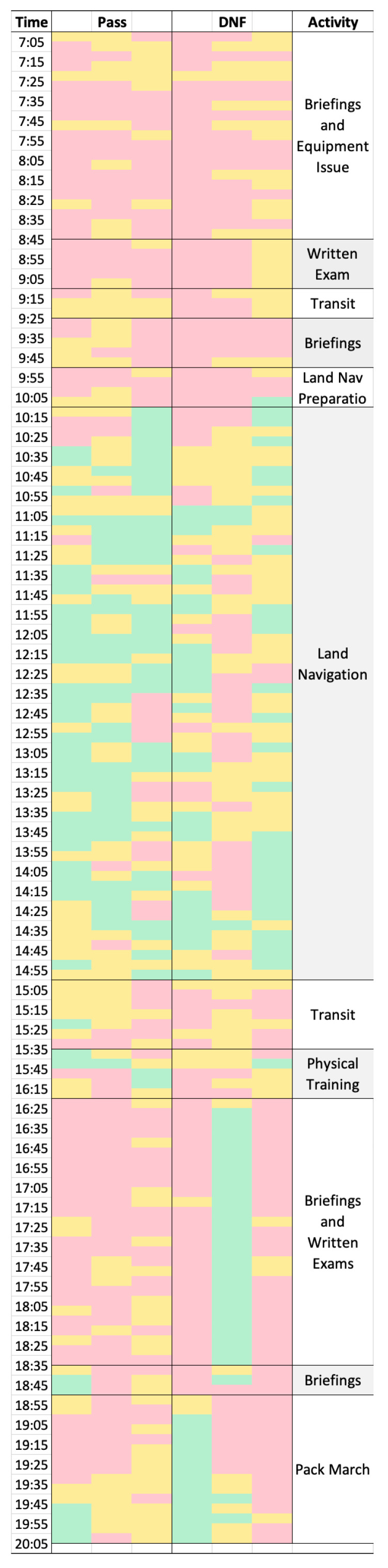
Heat map visualization of candidate pRR50 values in 5 min short-term blocks. PTG Candidates, n = 6. Red regions indicate values below ½SD of the participant’s mean, yellow regions indicate values within ± ½SD of the participant’s mean and green regions indicate values above ½SD from each participant’s mean. The furthest right column indicates activities and changes in activities throughout the recording period. The ‘Pass’ column indicates the three candidates that continued the selection course beyond the time period presented. DNF (did not finish) indicates the three candidates that withdrew during the pack march.

All collected data fell within the ‘low’ range for artifact presence (<20%) [40]. Three candidates withdrew during the pack march event. Two candidates withdrew the following day, with only one candidate ultimately completing the entire two-day course. During the data capture period considered in this study, temperatures ranged from 24.2–34.4 °C and relative humidity ranged from 60–75%. For every five minutes of recording, HRV was measured as the percentage of R-R intervals varying by at least 50 ms, yielding 153 consecutive short-term HRV analyses. The integration of these data into the finalized shaded heat map can be found in Figure 1. The column furthest right in Figure 1 provides information regarding the activity being completed at the time of HRV recording. Time of day is also provided for context in the column furthest left in Figure 1. Table 2 and Table 3 describe count data derived from the heat map (Figure 1) analysis. The quantitative values of interest from the heat map include the total amount of time each candidate had shaded for red and green, respectively, as well as the longest consecutive time period without change in shaded colour, representing prolonged high (green) or low (red) HRV for the epoch.

Regarding the Mann–Whitney U-tests, there were no statistically significant results from any variables (raw HRV data, count data totals, and count data consecutive sequences). Of the logistic regression analyses performed, only one was successful. The regression model that was statistically significant (χ^2^ (6) = 8.318, *p* = 0.004) was for count data of 5 min blocks with pRR50 intervals above ½SD from the mean value during the beginning of the selection through to the end of the land navigation exercise (0700–1430). That model correctly identified 100% of candidates that passed the pack march and 100% of candidates that failed the pack march. The resultant sensitivity of high HRV predicting that a candidate would pass the pack march event that followed was 1.0, and the specificity was also 1.0.

The heat map visualization of candidate’s pRR50 values illustrates the fluctuations in HRV during 13 h of load carriage activities (Figure 1). In a qualitative consideration of the heat map, all candidates demonstrated an initial period of minimal variability and values below ½SD of their individual mean values (areas shaded red). This lasted approximately three hours (07:05–10:05). Some recovery is noted once the candidates were underway and commencing the first land navigation activity, beginning at approximately 10:05 (areas shaded yellow and green). Values generally deteriorated again as the first load carriage activity ended. Participants then proceeded to another period of briefing and exams indicated at 18:50.

## 4. Discussion

The primary aim of this research was to determine if HRV data obtained during specialist police selection load carriage activities could be processed into an accessible format for end-users, and if the data could predict attrition. The capture, processing, and description of the data of an initial six-member cohort was completed to provide useful individualised feedback to unit selection course staff and leadership. Specifically, a logistic regression model was able to predict those that would complete the first day of selection from those that would not. In even a small cohort, with less HRV data than the extant literature would suggest is necessary [52], this model was statistically significant and could be utilized in future selection cohorts to identify at-risk candidates before their risk of elimination from selection. The captured data, their description, and presentation were analysed and interpreted to provide the greatest practical value to selection staff personnel and key stakeholders within the tactical police profession. The novel heat map approach (Figure 1) to visualise HRV data potentially demonstrates the levels of physical and psychophysiological stress each individual candidate experienced during selection course events and allowed concurrent monitoring of all candidates. While these results are certainly promising, this report may best be interpreted as an effective proof of concept but with a need for replication with a larger sample size.

Overall, the heat map approach to HRV interpretation identified the components of assessment that were the most taxing, as indicated by lengthy periods of low HRV, particularly those periods of pRR50 below ½SD of each candidate’s mean value. This process also allowed for the most powerful regression model to be developed for predicting attrition; the raw HRV data were not statistically significantly predictive, nor was low HRV. The initial observed period of diminished variability may have been due to several factors (namely circadian rhythm influences) [53,54,55]. However, the most likely reasons can be postulated to be latent exercise-induced sympathetic dominance from aerobic endurance testing occurring prior to the start of the presented data [56], or anticipatory stress [32,57]. Environmental considerations should also be contemplated, as previous research has determined that, even in highly trained individuals, high heat and humidity exposure may reduce parasympathetic and (or) enhance sympathetic modulations [18], likely also decreasing the pRR50 percentage in this sample. Regarding anticipatory stress, incomplete information and uncertainty were deliberately imposed by the selection staff as an important element of trainability assessment. A body of relevant literature is emerging, assessing relationships between stress, HRV, and uncertainty in which stress imposed by uncertainty acts to impair the physiological mechanisms that promote higher HRV [58,59,60]. While one investigation specific to the law enforcement profession did not find significant relationships between intolerance of uncertainty and physiological response as measured specifically by mean heart rate [61], HRV was not specifically analysed; only HR was reported in the referenced work, and therefore the greater sensitivity provided by HRV may be crucial in this assessment strategy. Indeed, HRV analysis, potentially including heat map interpretations, may yield more information than HR alone [38].

The establishment of this plausible relationship between uncertainty intolerance and its downstream effects on HRV may be of high value to selection staff and unit leadership. Selection course staff may aim to preferentially select candidates with either minimal psychophysiological response to uncertainty, or an ability to continue functioning effectively despite an elevated psychophysiological response. In the present reported data, all candidates, except for one candidate that did not finish the pack march, demonstrated prolonged decreased HRV during the written examination and briefing period following the land navigation exercise. This may highlight the impact of cognitive load on HRV, decreasing values and potentially impairing recovery. For this candidate, it is possible that the transition from a hot outdoor environment to a cooled indoor environment contributed to this elevation in HRV to a greater extent than the other candidates due to the greater stress experienced during the land navigation event, as evidence by greater periods below ½SD of mean long-term pRR50 relative to peers. It is also possible that this participant entered a heightened parasympathetic state due to overstress. Indeed, a U-shape distribution for describing HRV and disease risk has been proposed in the psychiatric literature [25]. The sports performance literature also supports high HRV in a context in which it is not expected (e.g., not a resting or post-prandial state) indicating performance deterioration as well [62].

Regarding the load carriage events, all candidates with the except of one that did not complete the pack march exhibited minimal impact from the pack march event relative to the previous exercises, and even improved in values during this time. This may indicate some capacity for recovery in spite of continued selection activities. The potential recovery period noted once the first load carriage task commenced may reflect a natural response to resolution of uncertainty; the candidates knew, once underway, the parameters of their task and were familiarized with it. This premise is supported by the recent psychiatric literature that determined depressed HRV to be indicative of high stress and anxiety states [25]. Indeed, as typical in these specialist populations [14], the candidates were of generally high aerobic fitness (mean MSFT Score: 84.2 ± 7.2 shuttles or approximately level 10.1), and the physical component alone may not have been a substantially taxing factor for this assessment. The re-emergence of uncertainty intolerance may have influenced the decreased HRV values, in combination with fatigue, noted during the initiation of second load carriage event [32,59]; the candidates were not informed the time or distance needed for success. Further research in this domain may provide more certainty regarding how these relationships manifest in this unique population. Finally, the first candidate to withdraw did not appear to have a similar increase in pRR50 percentage with the commencement of the second load carriage navigation event, indicating little recovery or return of dynamic adaptive regulation. This can be seen as continued regions of red and yellow in the one region of the DNF column in Figure 1, contrasting with the data trends observed in the other candidates beginning at approximately 1855. Another period of potential additional anticipatory stress may have occurred as the next load carriage event began.

While there were no statistically significant differences in the total number of short-term analyses below ½SD of mean long-term pRR50 threshold between the candidates who completed the final load carriage event and those who failed to complete the event (or continue after the event), the candidate that had the shortest consecutive sequence of pRR50 values below ½SD of mean was candidate 4, who did pass the pack march. This distinction is important because optimal HRV is characterized by dynamic adaptive response—prolonged instances of limited variation of either atypically high or low values potentially indicate suboptimal stress response [31,52]. While low HRV did not appear to necessarily be indicative of success, time spent with high HRV was predictive of at least progressing into the second selection day, as demonstrated by the outcome of the logistic regression model.

Visible, outward demonstrations of difficulty with task completion may not necessarily be reflected by the individual’s external environment. For example, while a candidate may not present as struggling by outward appearance, they may nonetheless be subject to high stress. This is of importance to selection staff who are assessing how each individual manages stress in a given situation. As such, individualised heat maps of candidate stress may provide highly valuable information to selection staff and unit leadership. In addition, identifying those candidates who are failing to recover may allow for adjustments in the activity to be made if needed, thereby helping to prevent the high selection failure rate described in this study and elsewhere [20,42,63].

### Limitations

This study was not without its limitations. As discussed above, while the sample size was not unusual for the population of interest, it nonetheless may diminish the generalizability of the reported findings. The approach provided here may not prove effective outside of the organisation within which this study was conducted. Further investigations with either multiple cohorts or larger cohorts are warranted to verify if modelling HRV data in the manner reported is sufficiently robust for wider application. Additionally, while the heat map visualization provided additional insight for unit leadership, the data could not be obtained, aggregated, interpreted, and visualized before attrition events occurred. Future studies or selection course operators may consider utilizing real-time HRV monitoring, or more frequent data processing to acquire this valuable data ahead of attrition occurrences. The data reported here demonstrate the potential of HRV as a robust biomarker that may hold value, but the time from data acquisition to reporting requires additional expediency.

## 5. Conclusions

The assessment and heat mapping of HRV can provide additional insight to selection staff and unit leadership during selection courses where the performance of each individual is critically analysed. The processing of HRV data using this approach allowed for a statistically significant regression model predicting attrition to be generated that was not possible with raw HRV values alone. This monitoring and data processing approach can be used to identify poorly performing candidates, and with future research may allow for intervention before medical attention is necessary. These data and approach may also support the objective identification of exceptional performers. Further, those who continue to demonstrate competence despite potential psychophysiological overstress as measured by HRV can also be identified. As the ability to suppress discomfort and function as part of a team is essential for the specialist police occupation, this additional information that can be provided within selection courses allows decision makers additional detail and objective, rather than subjective, means of holistic candidate analysis. While further research is needed to verify the objective relationships between HRV profile, performance, withdrawal from training, and other outcomes, this study demonstrates the unique utility of HRV and heat map generation of biosignals in specialist police selection.

## Figures and Tables

**Table 1:** Anthropometric, load carriage, and fitness data of Australian PTG candidates.

Value	Body Mass (Kg)	Height (cm)	Age (Years)	Land Nav Equipment Mass (Kg)	Pack March Equipment Mass (Kg)	BMI	MSFT (Level. Shuttles)
Mean	93.63	180.17	30.67	12.24	11.95	28.88	10.1
SD	8.83	5.98	2.94	0.14	1.32	2.92	0.5
Median	94.78	181.00	31.00	12.24	11.96	29.22	10.1
Range	20.50	21.08	14.00	8.00	0.20	8.47	1.9

Legend: body mass index (BMI), multistage fitness test (MSFT). Range is reported as the difference between maximum and minimum values.

**Table 2:** Count data: Total and longest consecutive time of HRV (pRR50) registered below ½SD of the participant’s mean.

	Total Time (min)	Consecutive Minutes
Finished Pack March (n = 3)	326.67 ± 5.77	56.67 ± 25.17
Did not Finish (DNF) Pack March (n = 3)	363.33 ± 57.95	116.67 ± 70.06

All values are presented as mean ± standard deviation.

**Table 3:** Count data: Total and longest consecutive time of HRV (pRR50) registered above ½SD of the participant’s mean.

	Total Time (min)	Consecutive Minutes
Finished Pack March (n = 3)	173.33 ± 30.14	38.33 ± 7.64
Did not Finish (DNF) Pack March (n = 3)	165.00 ± 22.91	80.00 ± 44.44

All values are presented as mean ± standard deviation.

## Data Availability

The datasets supporting the conclusions of this article are not publicly available as data were obtained from a law enforcement agency, and as per research ethics provisions, individual participant data cannot be released without a specific request to, and approval from, the sponsoring agency. To make a request, or for further information, please contact Dr. Rob Orr, Bond University Tactical Research Unit; rorr@bond.edu.au.

## Supplementary Materials

The following supporting information can be downloaded at https://www.mdpi.com/article/10.3390/healthcare11192677/s1. Figure S1. Histogram plots of each participant’s pRR50 values for each 5 min HRV analysis.
