# Supplementary material for: Heart Rate Variability Assessment of Land Navigation and Load Carriage Activities in Specialist Police Selection
Source: Healthcare (Basel). 2023 Oct 3;11(19):2677. doi: 10.3390/healthcare11192677 (PMC10572114; doi:10.3390/healthcare11192677)
Supplement: Supplementary file 1 [file healthcare-11-02677-s001.zip › healthcare-2570029-supplementary.pdf]

Supplementary File

Presented below are histograms visualizing the distribution of each participant's HRV values for each 5min HRV analysis (153 in total).

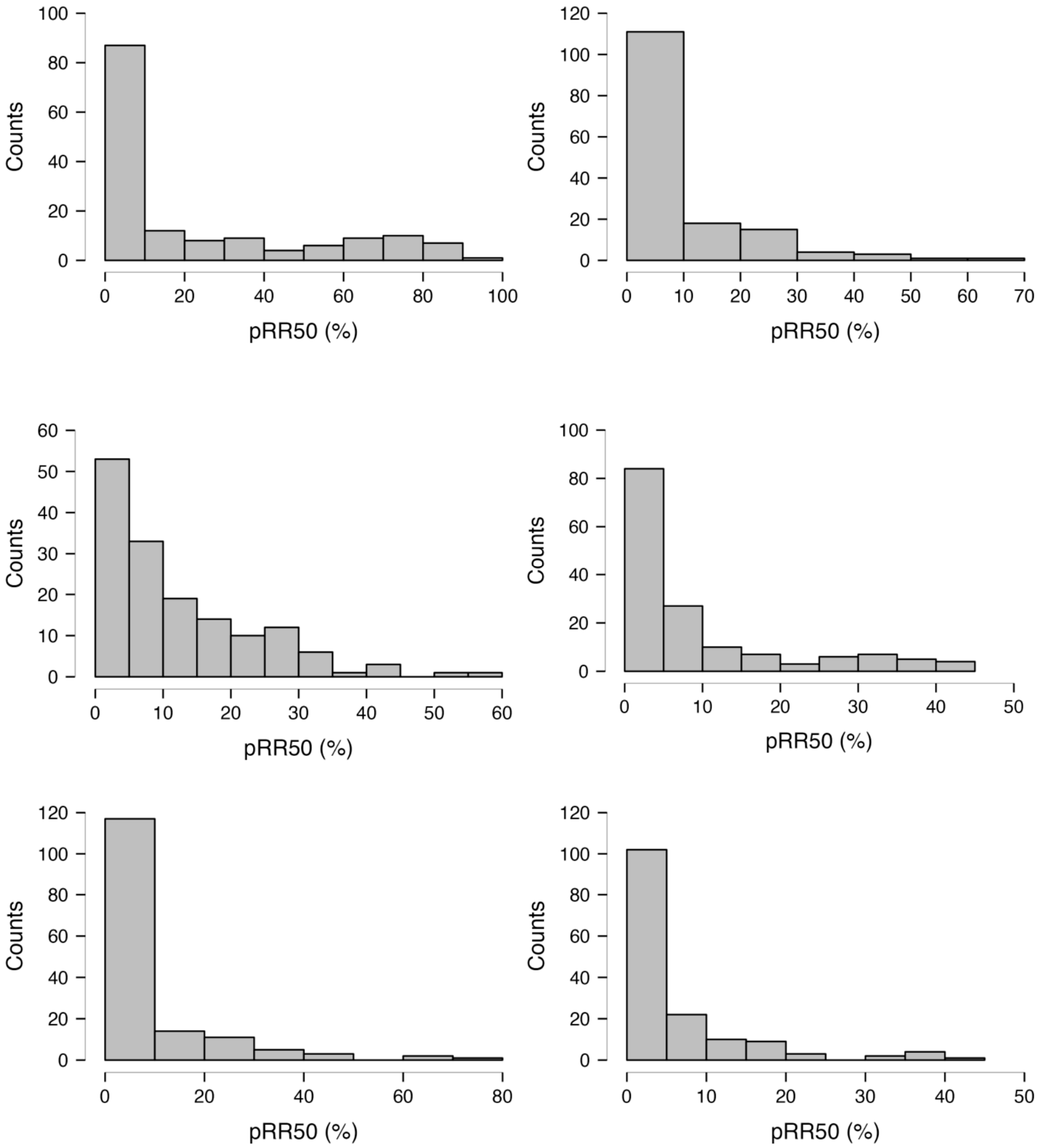

Figure S1. Histogram plots of each participant's pRR50 values for each 5 min HRV analysis.
